# Optimized delivered oxygen concentration of a novel pediatric oxygen mask: a bench simulation study

**DOI:** 10.1186/s12871-025-03598-7

**Published:** 2026-01-12

**Authors:** Yueh-Yin Chen, Nai-Ju Chan, Ya-Tin Lin, Tzu-Chun Kan, Pei-Jung Chang, Chun-Chun Hsu

**Affiliations:** 1 School of Respiratory Therapy, College of Medicine, Taipei Medical University, Taipei, 110 Taiwan; 2 Master Program in Thoracic Medicine, School of Respiratory Therapy, College of Medicine, Taipei Medical University, Taipei, 110 Taiwan; 3 Graduate Institute of Medical Sciences, College of Medicine, Taipei Medical University, Taipei, 110 Taiwan; 4 Department of Physiology, School of Medicine, College of Medicine, Taipei Medical University, Taipei, 110 Taiwan; 5 Graduate Institute of Metabolism and Obesity Sciences, College of Nutrition & TMU Research Center for Digestive Medicine, Taipei Medical University, Taipei, 110 Taiwan; 6 Nutrition Research Center, Taipei Medical University Hospital, Taipei, 110 Taiwan; 7 Genomics Research Center, Academia Sinica, Taipei, 115 Taiwan; 8 Research Center of Digital Oral Science and Technology, College of Oral Medicine, Taipei Medical University, Taipei, 110 Taiwan; 9 Graduate Institute of Manufacturing Technology, National Taipei University of Technology, Taipei, 106 Taiwan; 10 Division of Pulmonary Medicine, Department of Internal Medicine, Taipei Medical University Hospital, Taipei, 110 Taiwan

**Keywords:** Pediatric, Oxygen therapy, Breathing model, Medical device, Performance evaluation

## Abstract

**Background:**

Children have unique respiratory physiology, including low tidal volume, high respiratory rate, and low functional residual capacity, which often leads to dilution of inspired oxygen when using conventional oxygen masks. The SentriO Oxy™ mask is designed to provide a consistent, high oxygen concentration even at relatively low flow rates. This study evaluated its performance under simulated pediatric breathing conditions.

**Methods:**

A pediatric breathing simulator was used to test three respiratory rates [20, 30, 40 breaths per min (BPM)] and two tidal volumes (75 and 150 mL) at oxygen flow rates of 5 and 10 L/min, using either 600-mL or 1000-mL reservoirs. Oxygen concentration was continuously measured with a calibrated FlowAnalyser™. Five repeated trials were performed for each condition, and the maximum oxygen concentration was used for analysis.

**Results:**

With a 600-mL reservoir at 5 L/min, oxygen concentration decreased with higher respiratory rates. The 1000-mL reservoir delivered higher and more stable oxygen levels, particularly at 20 and 30 BPM. Increasing flow to 10 L/min improved oxygen delivery across reservoir sizes. The SentriO Oxy™ maintained ~ 80% oxygen concentration across most simulated pediatric breathing conditions.

**Conclusions:**

In this bench simulation study, the SentriO Oxy™ provided high, stable oxygen concentrations across different pediatric breathing patterns, supporting its potential clinical usefulness in both acute and long-term pediatric oxygen therapy.

## Introduction

Hypoxemia is a common and life-threatening sign in children suffering from acute respiratory conditions such as pneumonia, bronchiolitis, asthma attacks, and respiratory distress syndrome [1–3]. While oxygen therapy is the primary treatment, its effectiveness is often compromised by children’s distinct respiratory physiology. Children tend to breathe faster with smaller tidal volumes, which can dilute the inspired oxygen when using standard delivery devices. Typical nasal cannulas and face masks deliver oxygen based on flow rate, but at increased breathing rates, mixing with ambient air can significantly decrease the inspired oxygen fraction [4]. This underscores the necessity for advanced oxygen delivery systems capable of providing high, stable oxygen concentrations suitable for various pediatric respiratory conditions.

Although COVID-19 highlighted the importance of reliable oxygen delivery, the clinical challenge of maintaining stable oxygenation in children extends far beyond the pandemic [5]. Young children, particularly neonates and infants, have distinct anatomical and physiological characteristics that complicate oxygen delivery. They breathe with small tidal volumes and high respiratory rates [6] and have a highly compliant chest wall that provides limited opposition to the lungs’ inherent tendency to collapse [7]. Consequently, their functional residual capacity is low and may approach the alveolar closing volume, making them more vulnerable to airway closure and oxygen desaturation under stress [2, 8]. These unique features underscore the need for oxygen delivery systems capable of maintaining stable oxygen concentrations despite rapid and variable pediatric breathing patterns [8, 9].

Devices used in conventional pediatric oxygen therapy were limited by oxygen-flow requirements for humidification or by discomfort with noninvasive positive-pressure ventilation, resulting in variability in oxygen delivery [10]. The SentriO Oxy™ is a newly developed mask system designed to improve oxygen delivery efficiency by maintaining consistent and high oxygen concentrations even at low flow rates. This feature could be especially useful in pediatric care, where conserving oxygen and ensuring therapeutic effectiveness are both important. However, there is still limited evidence about its performance in pediatric respiratory settings.

To address this gap, we conducted a bench study using a simulated pediatric lung model to assess the efficacy of the SentriO Oxy™. Specifically, we investigated its ability to deliver high oxygen concentrations across a range of respiratory rates, tidal volumes, and flow rates that mimic pediatric breathing patterns. Findings from this study could offer valuable insights into the potential of SentriO Oxy™ as a new and effective oxygen therapy option for children with acute hypoxemic respiratory illnesses.

## Materials and methods

### Experimental setup

**Fig. 1:**
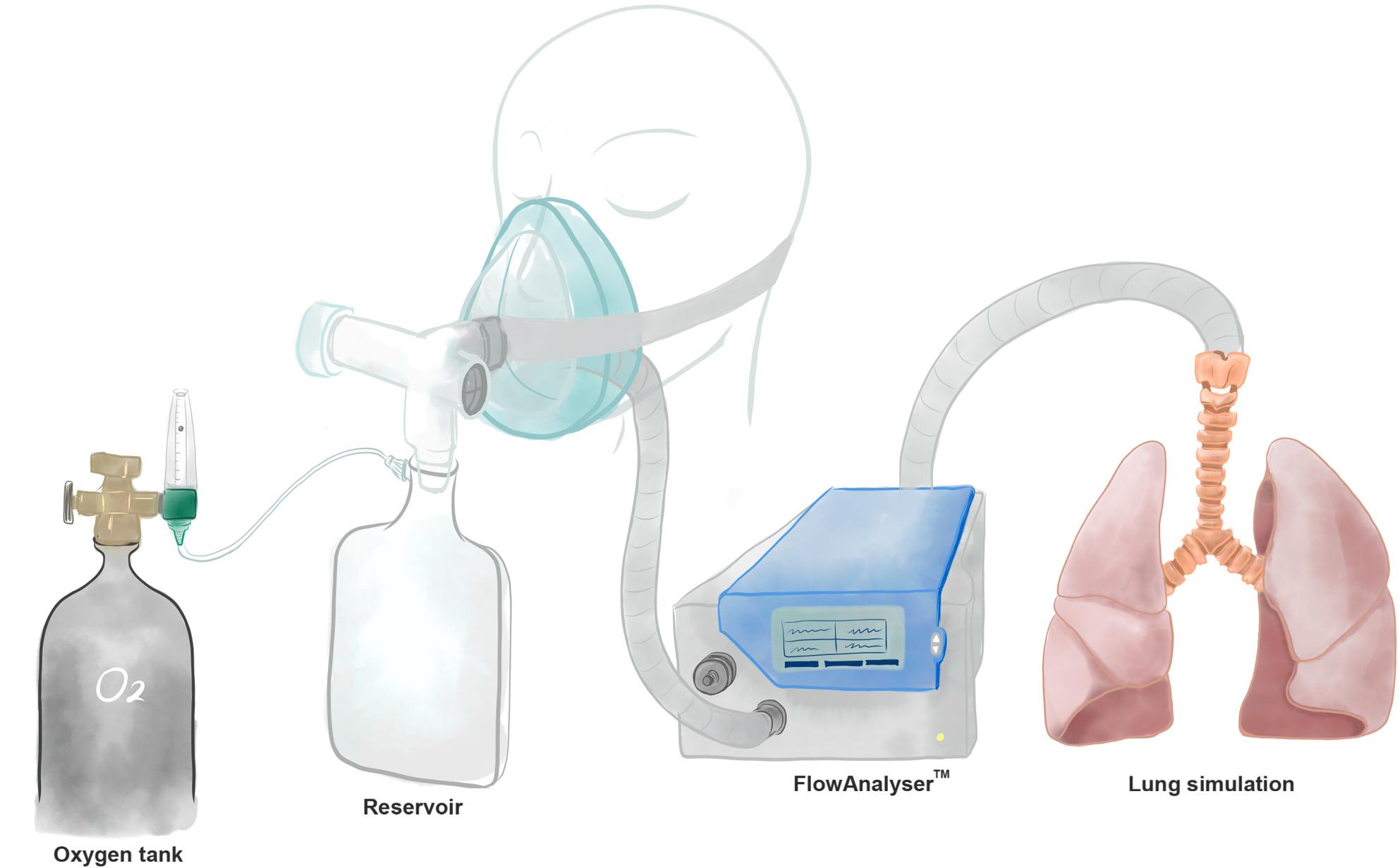
Experimental apparatus used to measure oxygen concentration in a simulated lung model. The device consisted of four main assemblies: an oxygen tank with a compensated flowmeter providing oxygen between 1-15 L/min, a facial mannequin simulating the patient-mask interface, the flow analyzer (FlowAnalyser^TM^ PF-300, IMT medical) accurately detecting the flow rate and oxygen concentration of the incoming and outgoing airflow, and a simulator mimicking the respiratory breathing pattern and ventilation

Medical-grade oxygen was supplied from a pressurized cylinder through a calibrated flowmeter and directed into either a 600-mL or 1000-mL reservoir bag before entering the SentriO Oxy™ mask (Fig. 1). The mask was connected to a pediatric breathing simulator (Michigan Instruments, Grand Rapids, MI, USA) configured to replicate the respiratory mechanics of infants and young children. Lung compliance was set to 4.0 mL/cmH_2_O and airway resistance to 50 cmH_2_O/L/s, values that fall within the physiologic ranges reported for infants and young children. The simulator generated tidal volumes of 75 mL or 150 mL at respiratory rates of 20, 30, or 40 breaths per min (BPM) to mimic the breathing conditions of infants and young children.

### Oxygen measurement

Oxygen concentration was continuously monitored at the inspiratory limb using a FlowAnalyser™ PF-300 (IMT Analytics AG, Buchs, Switzerland). The oxygen sensor was positioned 5 cm upstream of the mask-simulator interface and sampled oxygen concentration at 100 Hz, allowing breath-by-breath assessment. The FlowAnalyser™ was calibrated prior to each experiment using a certified calibration gas in accordance with the manufacturer’s protocol. Briefly, 100% oxygen was applied to the FlowAnalyser™ PF-300 at 20–30 L/min for 75 s, followed by ambient air (0% oxygen) at the same flow and duration.

### Experimental protocol

Oxygen flow rates of 5 L/min and 10 L/min were evaluated across all combinations of reservoir size, respiratory rate, and tidal volume. Each condition was allowed to stabilize for 30 s before data acquisition. Five independent trials were performed per condition, each lasting 3 min, during which all breaths were recorded. Based on prior oxygen delivery studies and preliminary testing, maximum oxygen concentration was selected as the primary outcome because it reflects the peak inspiratory oxygen fraction and minimizes variability caused by breath-to-breath mixing.

### Statistical analysis

Data are presented as mean ± SEM. Group comparisons involving respiratory rate, tidal volume, reservoir size, and flow rate were analyzed using one-way or two-way analysis of variance (ANOVA), followed by Tukey’s multiple-comparison *post-hoc* test. Statistical analyses were performed using GraphPad Prism version 10.5.0 (GraphPad Software, San Diego, CA, USA). A *P* value < 0.05 was considered statistically significant.

## Results

**Fig. 2:**
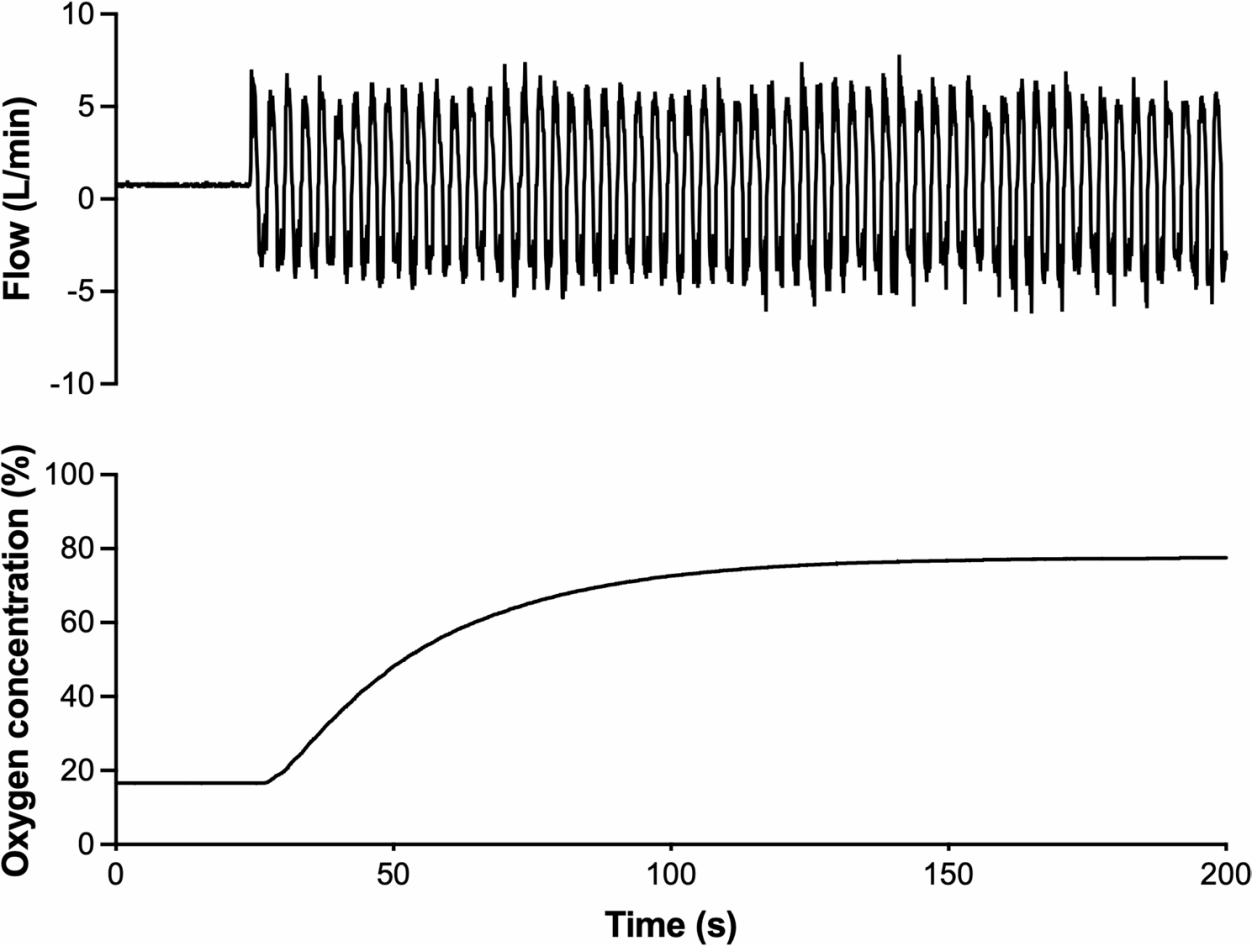
Experimental record showing oxygen concentration in the simulated lung model. In this model, oxygen was supplied from a tank with a flowmeter set at a constant flow rate of 5 L/min. The oxygen concentration provided by SentriO Oxy^TM^ was measured during continuous breathing simulated by the system (tidal volume: 75 mL; respiratory rate: 20 breaths per min). The oxygen concentration reached its maximum within 180 s

Figure 1 showed the experimental setup used to measure oxygen delivery in a simulated lung model that mimics pediatric breathing patterns. A representative recording of flow and oxygen concentration was provided in Fig. 2. During a continuous breathing trial (tidal volume: 75 mL; respiratory rate: 20 BPM; flow rate: 5 L/min), oxygen concentration rose rapidly and stabilized within approximately 3 min, remaining steady between breaths.

**Fig. 3:**
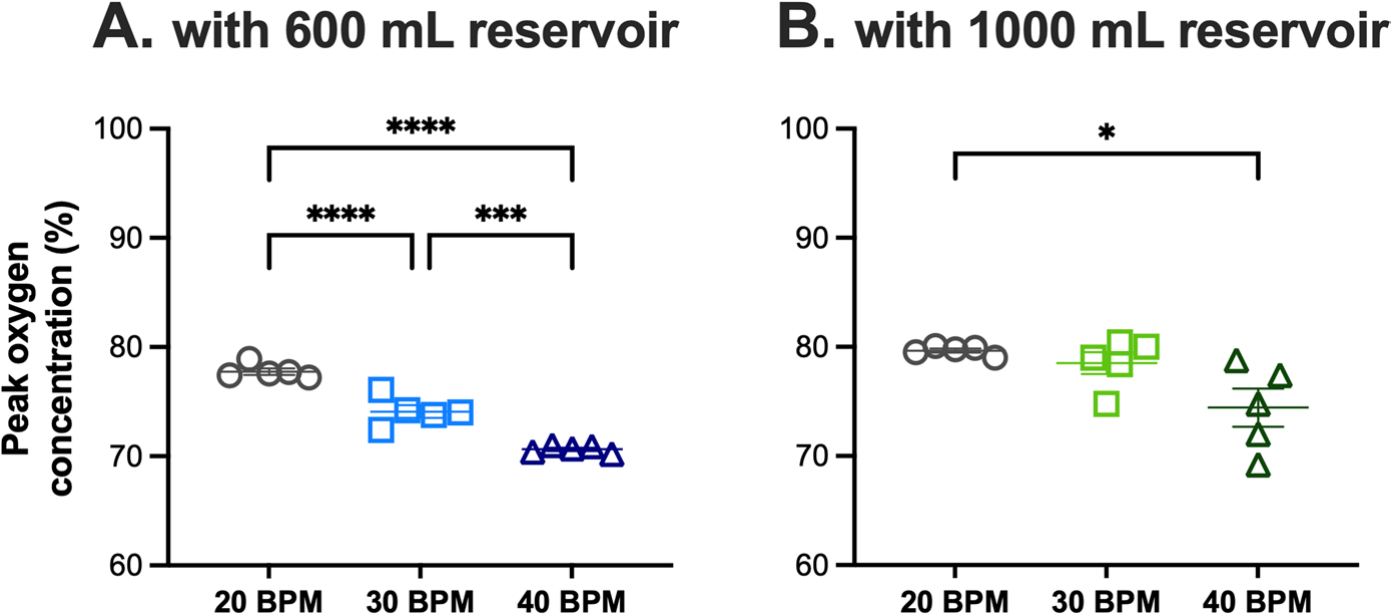
The peak oxygen concentration at different respiratory rates with 600 mL (**A**) or 1000 mL (**B**) reservoirs at a flow rate of 5 L/min and tidal volume of 75 mL.Oxygen concentration declined significantly with increasing respiratory rate when using the 600-mL reservoir, whereas the 1000-mL reservoir maintained higher levels with only a modest drop at 40 breaths per min (BPM). The 1000-mL reservoir delivered significantly higher oxygen concentrations than the 600-mL reservoir at 20 and 30 BPM. Data are presented as mean ± SEM from 5 trials of 3 min each. ****, *P *< 0.0001; ***, *P *< 0.001; *, *P *< 0.05 using one-way ANOVA with Tukey’s multiple comparisons test

At a flow rate of 5 L/min and a tidal volume of 75 mL, oxygen concentration decreased progressively as the respiratory rate increased with the 600-mL reservoir (Fig. 3A). In contrast, the 1000-mL reservoir produced higher and more stable oxygen levels across rates (Fig. 3B), with only a modest decline observed at 40 BPM. When comparing reservoir sizes directly, the 1000-mL reservoir consistently outperformed the 600-mL reservoir at low and moderate respiratory rates.

**Fig. 4:**
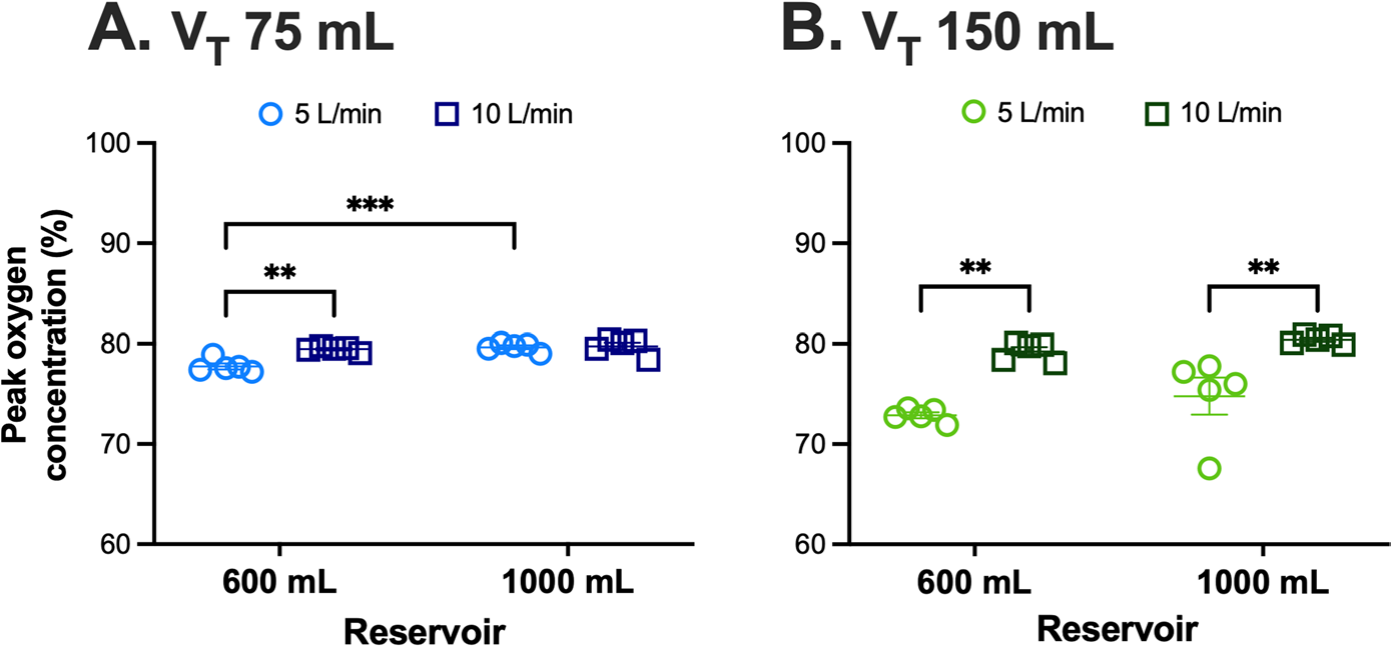
Effects of flow rate, tidal volume, and reservoir size on peak oxygen concentration at a respiratory rate of 20 breaths per min. **A** At a tidal volume of 75 mL, increasing flow from 5 to 10 L/min significantly improved oxygen concentration in the 600-mL reservoir, whereas changes in the 1000-mL reservoir were not significant. The 1000-mL reservoir delivered higher oxygen levels than the 600-mL reservoir at 5 L/min. **B **At a tidal volume of 150 mL, oxygen concentration was reduced at 5 L/min but increased significantly at 10 L/min for both reservoir sizes. Data are presented as mean± SEM from 5 trials of 3 min each. V_T_, tidal volume. ***, *P*< 0.001; **, *P *< 0.01 using two-way ANOVA with Turkey’s multiple comparisons test

At a respiratory rate of 20 BPM, we next examined the effect of flow rate and tidal volume (Fig. 4). With a tidal volume of 75 mL, increasing the flow rate from 5 to 10 L/min improved oxygen concentration with the 600-mL reservoir, whereas with the 1000-mL reservoir, the change was minimal (Fig. 4A). When tidal volume was increased to 150 mL, oxygen concentrations declined at 5 L/min for both reservoir sizes, suggesting increased inspiratory demand during larger tidal breaths (Fig. 4B). Increasing the oxygen flow to 10 L/min restored high oxygen concentrations in both the 600-mL and 1000-mL reservoirs (Fig. 4B). Cross-reservoir comparisons further demonstrated that the 1000-mL reservoir generally achieved higher oxygen concentrations than the 600-mL reservoir, particularly at lower tidal volumes and flow rates. Differences at 150 mL tidal volume were directionally similar but not statistically significant (Fig. 4).

Overall, in this pediatric breathing simulation model, the SentriO Oxy™ consistently maintained high oxygen concentrations (~ 80%) across a range of respiratory rates, tidal volumes, and flow conditions, demonstrating stable, efficient performance even under settings that typically challenge conventional oxygen delivery systems.

## Discussion

This bench study showed that the SentriO Oxy™ system effectively delivered high and stable oxygen concentrations in a pediatric breathing simulation model, even under challenging respiratory conditions. Conventional pediatric oxygen delivery devices are prone to dilute inspired oxygen, especially when respiratory rates are high and tidal volumes are small [6, 7]. In this study, we demonstrated that the SentriO Oxy™ consistently maintained oxygen concentrations within the 70 ~ 80% range, indicating it may overcome a significant limitation of traditional oxygen therapy in children with acute hypoxemia [6, 7].

Although the COVID-19 pandemic underscored the global importance of efficient oxygen delivery, the clinical challenge of providing stable oxygenation in children existed long before the pandemic and remains highly relevant [5]. The SentriO Oxy™ provides a resource-efficient solution by delivering high oxygen concentrations at relatively low flow rates, making it especially useful during acute outbreaks and in long-term pediatric care.

Pediatric oxygen delivery is highly influenced by respiratory pattern variations [6, 7, 11], and our findings highlight how reservoir size and flow rate interact with these physiological characteristics. In our simulations, the 1000-mL reservoir generally provided higher, more stable oxygen concentrations than the 600-mL reservoir across a range of respiratory rates in low-tidal-volume conditions. This pattern is likely related to the increased buffer volume, which reduces the entrainment of ambient air during rapid or higher-demand inspiratory cycles, a phenomenon well described in pediatric respiratory physiology [6, 7]. The difference became particularly evident at higher respiratory rates, when the smaller reservoir was more susceptible to dilution.

Tidal volume also plays an important role in shaping oxygen delivery performance [6, 12, 13]. When tidal volume increased to 150 mL, oxygen concentrations declined at 5 L/min in both reservoir configurations, suggesting that inspiratory demand exceeded the reservoir’s replenishment rate under low-flow conditions. Increasing the oxygen flow to 10 L/min compensated for this effect and restored high oxygen levels, consistent with the principle that pediatric oxygen systems must match inspiratory flow demand to avoid dilution [8, 9]. These observations underscore the importance of balancing reservoir volume and flow rate when managing oxygen delivery for children with varying breathing mechanics.

Our results parallel the trends reported by Chiang et al., who demonstrated in human airway measurements that the SentriO Oxy™ maintained high intratracheal oxygen concentrations across diverse breathing patterns [14]. Their findings provide physiological support for the reservoir-based trends observed in our bench model and reinforce the potential value of this system in stabilizing oxygen delivery under variable pediatric respiratory conditions.

Altogether, these findings demonstrate that the SentriO Oxy™ can maintain high inspired oxygen concentrations across a range of pediatric breathing conditions, highlighting its potential clinical value while underscoring the need for further validation in real pediatric patients. Further clinical studies will be essential to confirm the device’s performance in actual pediatric care settings.

### Limitations

This study has some limitations. First, the findings were derived from a bench simulation model, which, despite allowing precise control of respiratory parameters, cannot fully simulate real-life situations and reproduce the variability of pediatric breathing, including changes in inspiratory flow, airway resistance, or potential mask leakage [6, 7, 15, 16]. The assessment of patient acceptability and comfort with the SentriO Oxy™ requires further research. Second, oxygen concentration was the only measured outcome, and factors such as breathing effort, comfort, or interface tolerance were not evaluated. Even so, this simulation study provides important mechanistic insight into the performance of the SentriO Oxy™, and clinical investigations are needed to determine its effectiveness in real pediatric care.

## Conclusions

In this bench simulation study, the SentriO Oxy™ system reliably sustains high inspired oxygen concentrations across a range of pediatric breathing conditions. These findings provide strong preliminary evidence supporting its use as an alternative oxygen-delivery strategy for infants and young children, warranting clinical evaluation to confirm its performance in pediatric care settings.

## Data Availability

The data generated during the current study are available from the corresponding author on reasonable request.
